# Implementation Strategies to Enhance Safety-Net Hospitals’ Adoption of Screening, Brief Intervention, and Referral to Treatment for Opioid Use Disorder

**DOI:** 10.1007/s11606-025-09785-z

**Published:** 2025-08-04

**Authors:** Zoe Lindenfeld, Berkeley Franz, Cheyenne Fenstemaker, Alden Yuanhong Lai, Jose A. Pagan, Cory E. Cronin, Ji Eun Chang

**Affiliations:** 1 Edward J. Bloustein School of Planning and Public Policy, Rutgers University, New Brunswick, NJ, USA; 2 Heritage College of Osteopathic Medicine, The Institute to Advance Health Equity, Ohio University, Athens, OH, USA; 3 Department of Public Health Policy and Management, School of Global Public Health, New York University, New York, NY, USA; 4 College of Health Sciences and Professions, The Institute to Advance Health Equity, Ohio University, Athens, OH, USA

**Keywords:** care coordination, care transitions, hospitals, medication for opioid use disorder, opioid use disorder, substance use disorder

## Abstract

**BACKGROUND:**

To support adoption of Screening, Brief Intervention, and Referral to Treatment (SBIRT) for opioid use disorder (OUD), hospitals are likely to benefit from implementation strategies that are responsive to their unique environments. Yet there remains a gap in knowledge regarding which implementation strategies are needed to support SBIRT implementation and sustainment for OUD within safety-net hospitals.

**OBJECTIVES:**

To obtain expert consensus on the most effective implementation strategies to enhance the adoption of SBIRT for OUD in safety-net hospitals.

**DESIGN AND SETTING:**

A modified Delphi study, with two rounds of online surveys.

**PARTICIPANTS:**

Eighteen US-based experts from within the fields of OUD-focused health services research, addiction medicine, and emergency medicine.

**MAIN MEASURES:**

The primary outcome was consensus on 35 potential SBIRT implementation strategies, ranked on a 5-point Likert scale across three domains: Effectiveness, Feasibility, Impact on Equity. Consensus across respondents within both rounds was evaluated using the interquartile range. If the IQR was 1 or below on the 0 to 5 Likert scale, consensus was considered obtained. Items with a median value of 4 or higher were considered high priority.

**RESULTS:**

Following two rounds of ranking, consensus was achieved for all survey items. In total, 62.85% strategies (*n* = 22) were rated as High in Effectiveness, 20.0% (*n* = 7) were rated as High in Feasibility, and 11.42% (*n* = 4) were rated High in Impact on Equity. Seven strategies ranked high in two areas, with three—Identify and Prepare Champions, Identify Early Adopters, and Conduct Educational Meetings—ranked as highly effective and feasible.

**CONCLUSIONS:**

This consensus process provides strong support for implementation strategies that can be used to guide future practice and study. This work can encourage implementation of SBIRT for OUD within safety-net hospitals, and set the stage for future studies to evaluate the impact of different implementation strategies on patient outcomes following SBIRT.

## INTRODUCTION

Over the past two decades, rates of opioid use disorder (OUD) in the United States (US) have skyrocketed, causing significant morbidity and mortality. Although recent data demonstrates that overdose mortality declined in 2024, deaths remain high, with approximately 87,000 deaths due to drug overdose reported that year.^1^ In recent years, the risks associated with OUD—which include high rates of mortality and morbidity due to infectious disease transmission and wound-site infections—have been elevated due to the entry of potent and highly addictive substances into the drug supply, such as fentanyl and xylazine.^2^ While effective and evidence-based treatment for OUD exists, specifically medications for opioid use disorder (MOUD) such as buprenorphine, naltrexone, and methadone, fewer than 20% of individuals with an OUD receive MOUD each year.^3^ Strategies to improve access to MOUD are therefore critical.

Hospitals have a key role to play in addressing OUD and supporting access to MOUD. Hospitalizations for people with OUD are common, stemming from overdose and secondary infections such as endocarditis, hepatitis C, HIV, and skin and soft tissue abscesses.^4^ Because many people with OUD lack regular medical care, hospitalization is a crucial potential juncture to screen for OUD, initiate MOUD, and support transitions to outpatient care.^5,6^ One model is Screening, Brief Intervention, and Referral to Treatment (SBIRT), an evidence-based approach to assess the severity of substance use, provide a brief intervention to educate patients, and increase their motivation to reduce risky behavior,^7^ and referral to treatment, such as MOUD, for those needing more extensive treatment.^8^ The brief intervention of SBIRT can increase uptake of MOUD by increasing motivation to engage in OUD treatment in some individuals.^9^

Although evidence on the effectiveness of SBIRT for OUD is mixed,^10^ two studies have linked hospital-based SBIRT for OUD to positive patient outcomes including increased care engagement^11,12^ and reductions in substance use, signaling its utility in hospital settings.^12^ Yet, challenges to SBIRT implementation remain, especially in resource-constrained safety-net hospitals that have high rates of OUD-related admissions.^13^ Safety-net hospitals disproportionately serve rural patients, individuals with Medicaid insurance, and people from minoritized racial/ethnic groups. In these communities, safety-net hospitals play a critical role as anchor institutions, delivering healthcare services to populations with limited access to other treatment settings.^14^ Thus, supporting SBIRT implementation within safety-net settings is critically important to reduce existing disparities in access to care for OUD.

SBIRT implementation barriers include funding and reimbursement concerns, time constraints, the need for leadership buy-in and support, and limited knowledge and self-efficacy among staff.^15–18^ Effective external referrals and transitions for high-risk individuals from safety-net hospitals to community treatment services is a particularly challenging part of SBIRT to implement.^19^ To overcome these barriers, studies conducted in primary care settings have identified best practices for SBIRT models, which include having a practice champion, utilizing data/days systems, and developing partnerships with external providers.[16,,20] However, there remains a gap in knowledge regarding which implementation strategies are needed to support SBIRT implementation and sustainment for OUD within safety-net hospitals.

To support adoption of hospital-based SBIRT for OUD, safety-net hospitals are likely to benefit from implementation strategies that are tailored and responsive to their unique environments and multiple contextual levels, including individual-level behavioral change, organizational-level change,^21–24^ and structural/policy-level change factors.^22^ An implementation strategy refers to an intentional, systematic intervention designed to integrate evidence-based practices into work processes for sustained use.^25^ Yet, previous studies have not tailored implementation strategies to the barriers and facilitators to implementing SBIRT that are specific to safety-net hospitals.

To address this gap in knowledge, the present study sought expert consensus on the most effective implementation strategies to enhance the adoption of SBIRT for OUD in safety-net hospitals. We did this through a Modified Delphi process adapted from the RAND UCLA appropriateness method, a well-known approach for establishing expert consensus.^26^ Specifically, we asked participants to rank potential implementation strategies designed to facilitate the adoption of SBIRT for OUD in safety-net hospitals according to three criteria: feasibility of adoption, effectiveness, impact on equity. The goal of this study was to inform a ranking of the most high-yield implementation strategies to increase the adoption of hospital-based SBIRT for OUD in communities where access to MOUD treatment is currently limited.

## METHODS

### Delphi Panel Recruitment

National experts from within the fields of OUD-focused health services research, addiction medicine, and emergency medicine were invited to participate. Experts were recruited if they had experience related to any of the following: (1) delivering care to hospitalized patients with OUD; (2) implementing OUD programs in safety-net hospitals, either as a clinician or administrator; (3) conducting research focused on initiatives to address OUD within hospital settings; (4) authoring high-impact publications related to hospital-based OUD treatment and care transitions; and/or (5) serving on National Academies consensus committees. Potential experts that met the inclusion criteria were identified through multiple strategies, which included reviewing author lists of peer-reviewed publications and national reports, searching safety-net hospital websites, reviewing National Academies committee rosters, and examining lists of speakers at relevant practitioner and academic conferences, including the American Public Health Association’s Annual Meeting, the AcademyHealth Annual Research Meeting, and the Addiction Health Services Research Conference. Our aim was to include a range of participant expertise, including both health professionals and researchers, selected based on knowledge of the field. Potential participants each received invitations to participate by email. Participants were offered a stipend of $400 if they agreed to participate and complete all survey rounds. The identity of the participants was not shared with the other participants. The RAND/UCLA Appropriateness Method (RAM) manual for conducting expert panels recommends a minimum of 9 experts,^26^ and in total, the study team contacted 23 potential participants.

### Survey Items and Analysis

Implementation strategy selection was informed by qualitative research conducted by the study team that focused on the barriers and facilitators to initiating MOUD within safety-net hospital settings and subsequent linkage to outpatient care, and a review of the literature conducted on SBIRT implementation within hospitals. The qualitative component involved semi-structured interviews conducted between November 2022 and August 2023 with 27 participants, including hospital providers, staff, and administrators across four safety-net health systems (*n* = 22) and staff at community-based organizations (CBOs) with established referral partnerships with the hospitals in the study (*n* = 5). The interview guide focused on facilitators and barriers to implementing transitional opioid programs in safety-net hospitals and forming partnerships with CBOs. Interviews were audio-recorded and professionally transcribed. Five team members developed an initial codebook through independent coding followed by discussions to achieve consensus, after which two study team members coded the remaining transcripts. Lastly, one study team member conducted axial coding,^27^ in which associations between and within codes are examined, to identify detailed themes of barriers and facilitators related to identifying patients with OUD in safety-net hospitals, initiating MOUD treatment, and transitioning to ongoing treatment in the community. This approach allowed for the identification of themes that informed the selection of implementation strategies. A detailed description of the qualitative methods and findings is available in a separate publication.^19^ Using the results from the qualitative component and literature focused on SBIRT implementation, the team extracted common barriers and facilitators to SBIRT from the prior interviews and the literature, and mapped them to ERIC implementation strategies.^21^ Two study members completed the mapping process to ensure reliability and completeness, with coding discrepancies resolved through discussions with the full study team. Following this process, the study team selected 35 potential implementation strategies for inclusion in the Delphi process.

The Delphi process included two rounds of surveying expert participants. In the first round, participants were asked about their demographic characteristics (gender identity, race/ethnicity), and professional characteristics (role, geography of clinical care/research, years of experience in OUD care or research, affiliation with safety-net organizations, and prior experience implementing SBIRT). Participants were then asked to rank 35 potential SBIRT implementation strategies (see Table 1) according to three domains: (1) Feasibility of adoption—how practical it is to implement these strategies in safety-net hospital settings; (2) Effectiveness—the expected effectiveness of this strategy in improving referral to treatment linkages; and (3) Impact on equity—the extent to which this strategy addresses and reduces disparities in access to care and treatment for OUD. We prioritized these domains as implementation criteria given that these areas are critical in safety-net hospitals, where resource constraints and patient population health needs, including health disparities, must be addressed simultaneously. While other implementation constructs such as reach, adoption, and sustainability are also critical, our decisions reflect the considerations relevant to early-stage implementation in low-resourced settings.^28,29^ Participants were asked to rank the 35 strategies across all three domains, which totaled 105 survey items. Ranking for each domain was conducted on a Likert scale ranging from 1 (low) to 5 (high). Implementation strategies were presented to participants according to six overarching categories: Category 1, Coalition Building and Stakeholder Involvement (strategies #1–9); Category 2, Education and Training (strategies #10–18); Category 3, Data and Feedback (strategies #19–22); Category 4, Tailoring to Local Context (strategies #23–27); Category 5 Technical and Financial Support (strategies #28–32), and Category 6, System Changes (strategies #33–35). Table 1 presents the definition of each strategy in an SBIRT context.

The survey for Round 1 was distributed to 20 experts on 10/15/2024 and active for 3 weeks. Consensus across respondents within the first round was evaluated using the interquartile range (IQR) calculated for the median value for each item/domain ranked. If the IQR was 1 or below on the 0 to 5 Likert scale, consensus was considered obtained.^30^ Using the median value for each strategy, we also assessed the importance of each strategy; items with a median value of 4 or higher were considered high priority, items with a median value of 3 were considered to be of moderate priority, and items with a median value of 2 or below were considered low priority.

The Round 2 survey was distributed to experts on 12/3/2024 and active for 3 weeks. For the second round, participants were provided with anonymous aggregated data of all measures identified from Round 1, including the median value for each item and whether consensus was achieved.^31^ Participants were asked to rate each measure that did not achieve consensus in Round 1 using the Likert scale presented in the initial survey. A flowchart of the Delphi ranking process can be found in Appendix Fig. 1.

Analyses were performed using STATA SE 18 and Microsoft Excel. This study was approved by the Ohio University Institutional Review Board.

## RESULTS

### Delphi Participants

Of the 23 invited recipients, 20 accepted the invitation to participate. Of those, 2 dropped out—one prior to the start of Round 1 and one after partial completion of Round 1. Characteristics for the 18 participants who completed both survey rounds are presented in Table 2. There were more female participants (*n* = 13, 72%) than male (*n* = 4, 22%) and non-binary (*n* = 1, 5%) participants. The majority identified as White (*n* = 15, 83%), and worked in an urban setting (*n* = 15, 83%). On average, participants had 12 years of experience working in substance use disorder care or research (SD, 8.17). Most were physicians (*n* = 12, 67%) or worked as researchers (*n* = 12, 67%), with approximately half working as addiction specialists (*n* = 10, 56%). Our sample included one addiction social worker (*n* = 1, 6%). Under half our sample had experienced implementing SBIRT (*n* = 8, 44%), and most participants had an affiliation with a safety-net organization (*n* = 16, 89%).

### SBIRT Implementation Strategy Ranking

**Figure 1:**
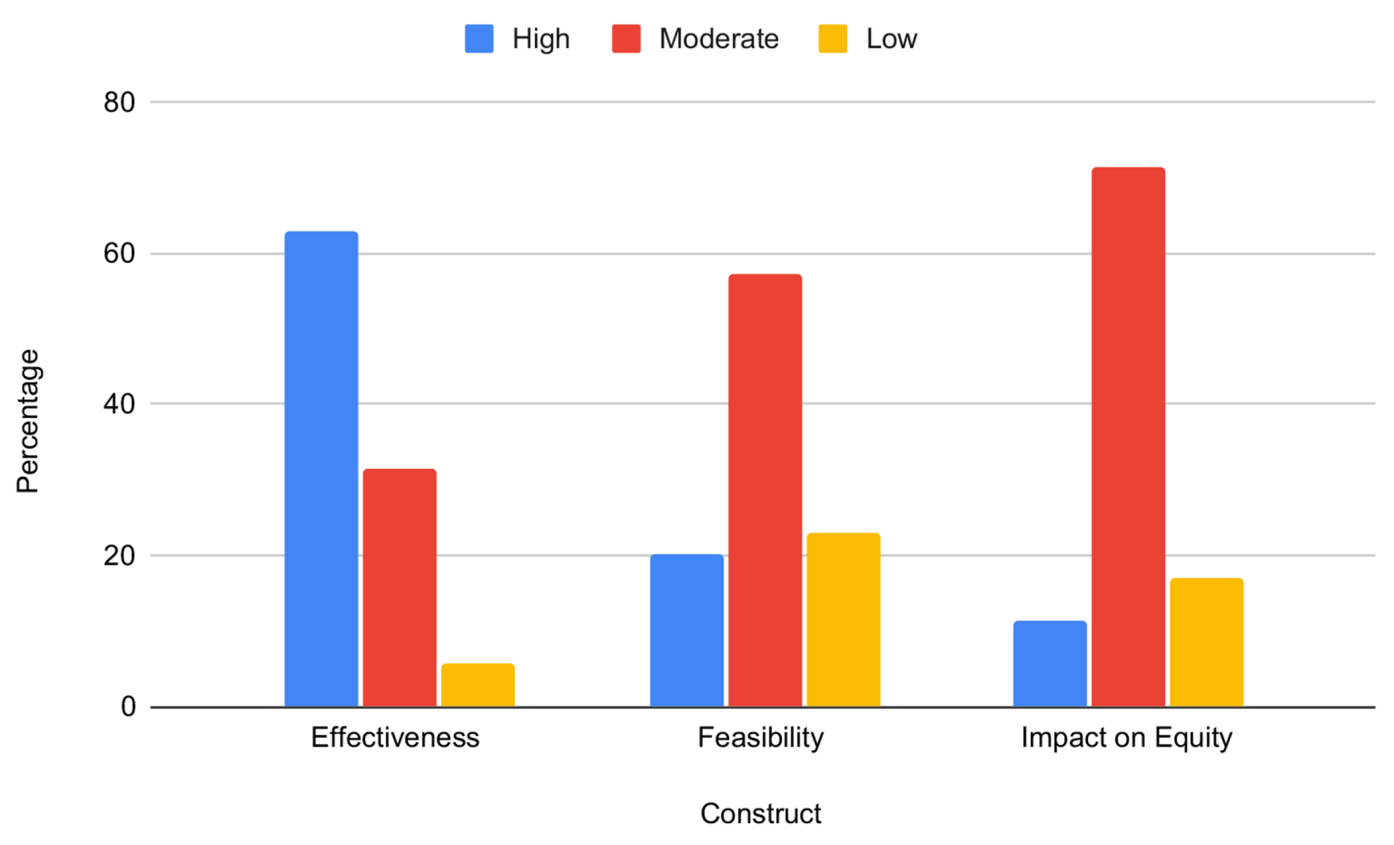
Percentage of strategies ranked as High, Moderate, and Low by domain (*n* = 35).

The 35 SBIRT implementation strategies, and their final rating on Effectiveness, Feasibility, and Impact on Equity, are presented in Table 3. In Round 1, consensus was reached on 56 items, and in Round 2, consensus was reached on the remaining 49 items. In total, 62.85% strategies (*n* = 22) were rated as High in Effectiveness, 20.0% (*n* = 7) were rated as High in Feasibility, and 11.42% (*n* = 4) were rated High in Impact on Equity (Fig. 1).

Of the nine strategies presented in Category 1, 77.77% (*n* = 7) were rated High in Effectiveness, 33.33% (*n* = 3) as High in Feasibility, and 22.22% (*n* = 2) as High in Impact on Equity (Table 3). Two strategies (Identify and prepare champions, Identify Early Adopters) were rated as High in both Effectiveness and Feasibility, and two (Build a Coalition, Involve patients/consumers and family members) were rated as High in both Effectiveness and Impact on Equity. Of the ten strategies presented in Category 2, 30% (*n* = 3) were rated High in Effectiveness, and 40% (*n* = 4) were rated as High in Feasibility; one strategy (Conduct Education Meetings) was rated as High in both Effectiveness and Feasibility. Of the four strategies presented in Category 3, 75% (*n* = 3) were rated as High in Effectiveness. In Category 4, of the five strategies presented, 80% (*n* = 4) were rated as High in Effectiveness, and 20% (*n* = 1) was rated as High in Impact on Equity; one strategy (Tailor strategies) was rated in High in both Effectiveness and Impact on Equity. Of the four strategies presented in Category 5, 75% (*n* = 3) were rated as High in Effectiveness. Of the three categories presented in Category 6, 66.66% (*n* = 2) were rated as High in Effectiveness, and 33.33% (*n* = 1) was rated as high in Impact on Equity; one strategy (Create new clinical teams) was rated High in both Effectiveness and Impact on Equity.

## DISCUSSION

This study aimed to establish expert consensus on the potential utility of various implementation strategies to support SBIRT implementation for patients with OUD in safety-net hospitals. This is a critical clinical area with limited prior empirical research. Following two rounds of Delphi ranking, we achieved consensus on all 35 strategies presented, 22 of which were ranked as highly effective by participants. Of these, three were also ranked highly feasible to implement. Yet, only four strategies were ranked as having a strong impact on equity. These findings have implications for both safety-net hospitals and non-safety-net hospitals seeking to implement SBIRT for OUD, though with access to varying amounts of resources to support implementation.

Important insights can be learned from the strategies ranked as both highly effective and feasible to implement—Identify and Prepare Champions, Identify Early Adopters, and Conduct Educational Meetings—as well as those strategies ranked by participants as being both highly effective and having a strong impact on equity—Build a Coalition, Involve patients/consumers and family members, and Create new clinical teams. Specifically, these findings indicate that obtaining buy-in from a range of stakeholders, including hospital leadership, clinical champions, and patient advocates, is critical to successful SBIRT implementation. This finding aligns with previous research focused on organizational change initiatives related to OUD. For example, previous research has found that allowing patients to drive conversations around their drug use and treatment needs is a key element of patient-centered care for patients with OUD, and can help dismantle mistrust and stigma between patients and providers.^32^ However, instituting these practices may require training for existing staff, or implementing new screening processes for hiring across the organization, in order to prioritize staff willing to implement these approaches.^33^ Additionally, while building community coalitions to address OUD and creating new clinical teams may not require significant organizational changes, these activities do require support from hospital leadership, as well as flexibility among staff to adopt new responsibilities (i.e., networking with external organizations and businesses), and shift into new roles or teams. Building community coalitions can also be an effective strategy for safety-net hospitals to improve access to MOUD, even if internal capacity to provide MOUD is limited due to staffing or financial constraints. By establishing partnerships with external organizations that offer MOUD or other more intensive treatment services, safety-net hospitals can help ensure that patients with OUD receive timely access to treatment, even if these services are not available within the hospital.^34^

Strategies ranked as highly effective with either moderate or low feasibility and impact on equity were those that required significant investments of time, resources, and training. These strategies included nearly all of the strategies within both the Data and Feedback and the Technical and Financial Support categories, all of which could enhance performance and communication among clinicians, and facilitate care transitions. However, these strategies also necessitate new, expensive data systems, as well as technological training for staff and physicians, who may lack advanced computer skills and be resistant to change.^35,36^ Similarly, the majority of strategies included in the Tailoring to Local Context category were considered effective; however, they would likely require significant time investments and implementation expertise to adopt. As such, while these strategies may not be feasible in lower-resourced settings such as safety-net hospitals, hospitals with more financial and staff resources that have not yet adopted SBIRT for OUD should consider these strategies as they move towards implementation.

This study is not without limitations. First, Delphi participants were a non-representative convenience sample based on authors’ knowledge and a review of the literature on SBIRT for OUD. While our sample included a range of professional roles, participants were mainly female, white, and based in urban settings, which likely have greater resources dedicated to OUD care; notably, the absence of Black/African American participants represents a limitation in the representativeness of our sample. Future work should aim to collect information from a more diverse set of participants, specifically those based in rural settings. Second, although we asked participants to rank each strategy independent of others, the implementation strategies presented are not mutually exclusive, and their impact likely depends on other available strategies, the institutional environment, and the surrounding community. Third, although participants were asked to suggest additional implementation strategies in addition to the ones presented in the survey, additional strategies were not suggested. This is potentially due to the relatively long length of the survey, which presented 35 potential implementation strategies to participants across three ranking criteria. Fourth, although the number of experts who participated in this study (*n* = 18) is higher than the minimum recommended by the RAM manual for conducting expert panels, the number of experts engaged is lower than in similar Delphi studies.^37^ Finally, this ranking is not exhaustive of all possible SBIRT implementation strategies, and we acknowledge that other strategies may still exist in the field.

## CONCLUSIONS

Despite these limitations, our approach of gathering and refining strategies through review of the scientific literature, and surveys with care providers, researchers, and addiction experts through an established consensus process provides strong support for these implementation strategies and can be used to guide future practice and study. In particular, this work can encourage implementation of SBIRT for OUD within hospitals and set the stage for future studies to evaluate the impact of different implementation strategies on patient outcomes following SBIRT. Specifically, studies should assess whether the use of certain implementation strategies—particularly those aimed at establishing networks and connections with external organizations—is associated with higher rates of patient transitions to ongoing treatment in the community following hospital discharge.

## Supplementary Material

Appendix Figure 1

The online version contains supplementary material available at https://doi.org/10.1007/s11606-025-09785-z.

## Figures and Tables

**Table 1:** Implementation Strategy Definitions

Category 1: Coalition Building and Stakeholder Involvement
Identify and prepare champions
Definition in SBIRT Context: Identify, prepare, and support individuals that provide SBIRT so that they can lead its implementation, get buy-in, and overcome resistance within the hospital
Identify Early Adopters
Definition in SBIRT Context: Identify and engage clinicians and staff who are already supportive of SBIRT to help lead and promote its adoption
Involve Executive Boards
Definition in SBIRT Context: Engage key hospital executives and/or governing board members in the planning and resource allocation for SBIRT to ensure institutional backing and sustainability
Build a Coalition
Definition in SBIRT Context: Form a coalition of local healthcare providers, community organizations, and other stakeholders to support and enhance SBIRT implementation
Involve patients/consumers and family members
Definition in SBIRT Context: Actively include patients and their families in developing, refining, and promoting the SBIRT program to ensure it meets patient needs and enhances patient-centered care
Conduct Local Consensus Discussions
Definition in SBIRT Context: Engage partners in discussions to reach consensus on how best to implement and adapt SBIRT to local needs and resources
Inform Local Opinion Leaders
Definition in SBIRT Context: Increase the level of SBIRT awareness and knowledge among key figures within the hospital to encourage their support and influence over their peers
Promote Network Weaving
Definition in SBIRT Context: Encourage connections between various hospital departments and external partners to facilitate better communication and collaboration on SBIRT
Obtain Formal Commitments
Definition in SBIRT Context: Secure commitments from hospital leadership and key departments to support and prioritize SBIRT implementation
**Category 2: Education and Training**
Develop academic partnerships
Definition in SBIRT Context: Collaborate with academic institutions to enhance the training, research, and implementation of evidence-based practices surrounding SBIRT
Recruit, designate, and train for leadership
Definition in SBIRT Context: Select and develop leadership within the hospital who can guide and support the SBIRT program
Conduct educational meetings
Organize meetings within the hospital to educate clinicians, administrators, and staff about SBIRT and discuss implementation strategies
Develop educational materials
Definition in SBIRT Context: Create tailored educational materials to facilitate learning and adoption of SBIRT within the hospital
Create a learning collaborative
Definition in SBIRT Context: Establish a collaborative network across hospitals to share insights and enhance SBIRT implementation within the health system
Conduct educational outreach visits
Definition in SBIRT Context: Conduct visits by SBIRT experts to partner organizations to educate and engage them in the referral process and the benefits of the SBIRT program
Use Train the Trainer strategies
Definition in SBIRT Context: Implement a Train the Trainer program to expand SBIRT knowledge and skills throughout the hospital efficiently
Conduct ongoing training
Definition in SBIRT Context: Organize continuous training opportunities for staff to stay updated on SBIRT protocols and best practices
Visit other sites
Definition in SBIRT Context: Organize visits for hospital staff to observe other institutions that have successfully implemented SBIRT, to learn and adopt best practices
Shadow other experts
Definition in SBIRT Context: Arrange for hospital staff to observe and learn from established SBIRT programs in other units
**Category 3: Data and Feedback**
Facilitate relay of clinical data to providers
Definition in SBIRT Context: Enhance systems to quickly and efficiently share relevant clinical data with providers, to inform and improve SBIRT processes
Use data experts
Definition in SBIRT Context: Employ data analysis experts to evaluate SBIRT’s effectiveness and guide its continuous improvement based on empirical evidence
Capture and share local knowledge
Definition in SBIRT Context: Document and disseminate successful practices and lessons learned from SBIRT implementation within the hospital
Develop and organize quality monitoring systems
Definition in SBIRT Context: Set up a comprehensive quality monitoring system across all SBIRT partnership entities to track effectiveness and ensure adherence to high standards of care
**Category 4: Tailoring to Local Context**
Assess for readiness and identify barriers and facilitators
Definition in SBIRT Context: Evaluate the hospital’s capacity for SBIRT implementation and identify obstacles and enablers within its environment
Tailor strategies
Definition in SBIRT Context: Customize SBIRT implementation tactics to align with the specific needs and conditions of the hospital
Conduct local needs assessments
Definition in SBIRT Context: Assess the specific needs of the hospital’s context and patient population to tailor the SBIRT program effectively
Promote adaptability
Definition in SBIRT Context: Encourage flexibility in how SBIRT is implemented in different hospital settings while maintaining core components of the program
Develop a formal implementation blueprint
Definition in SBIRT Context: Create detailed plans with partners outlining the steps, goals, and responsibilities for implementing SBIRT, ensuring all partners are aligned and committed
**Category 5: Technical and Financial Support**
Centralize technical assistance
Definition in SBIRT Context: Set up a central support system within the hospital to provide ongoing assistance with SBIRT implementation and troubleshooting
Access new funding
Definition in SBIRT Context: Secure financial resources to support the initiation and ongoing operations of the SBIRT program within the hospital
Fund and contract for clinical innovation
Definition in SBIRT Context: Leverage funding opportunities and contracts to encourage and support the use of SBIRT as a standard practice in the hospital
Develop resource sharing agreements
Definition inSBIRT Context: Formulate agreements between partners to share resources such as training materials, data, and expertise to support SBIRT implementation
**Category 6: System Change**
Change liability laws
Definition in SBIRT Context: Advocate for changes in liability laws to protect and encourage healthcare providers to implement SBIRT fully without fear of legal repercussions
Revise professional roles
Definition in SBIRT Context: Adapt and redefine the roles of healthcare professionals within the hospital to better integrate and support the delivery of SBIRT services
Create new clinical teams
Definition in SBIRT Context: Form multidisciplinary teams across partner organizations that specialize in SBIRT to provide comprehensive care and ensure continuity across different service points

**Table 2:** Delphi Panel Participant Self-Reported Characteristics (*n* = 18)

Characteristic	*N* (%)
Gender	
Male	4 (22%)
Female	13 (72%)
Non-binary	1 (6%)
Race/ethnicity^T^	
White	15 (83%)
Hispanic/Latinx	2 (11%)
Asian	1 (6%)
Middle Eastern or North African	1 (6%)
Professional role^T^	
Physician (MD/DO)	12 (67%)
Addiction specialist	10 (56%)
Researcher	12 (67%)
Social worker	1 (6%)
Primary geography of clinical care/research	
Urban	15 (83%)
Mixed urban/rural	3 (17%)
Prior experience implementing SBIRT	
Yes	8 (44%)
No	10 (56%)
Affiliation with safety-net organization	16 (89%)
Yes	2 (11%)
No	
Years of experience in substance use disorder care or research (mean and standard deviation)	12 (8.17)

*MD*, doctor of medicine; *DO*, doctor of osteopathic medicine.

T Options provided were not mutually exclusive

**Table 3:** SBIRT Implementation Strategy Final Rankings*

Strategy	Effectiveness	Feasibility	Impact on equity
**Category 1: Coalition Building and Stakeholder Involvement**			
Identify and prepare champions	High	**High**	Moderate
Identify Early Adopters	High	High	Moderate
Involve Executive Boards	Moderate	Moderate	Moderate
Build a Coalition	**High**	Moderate	**High**
Involve patients/consumers and family members	High	Moderate	High
Conduct Local Consensus Discussions	**High**	**Moderate**	Moderate
Inform Local Opinion Leaders	**Moderate**	**High**	**Low**
Promote Network Weaving	**High**	Moderate	Moderate
Obtain Formal Commitments	**High**	**Moderate**	Moderate
**Category 2: Education and Training**			
Develop academic partnerships	**Moderate**	**High**	**Moderate**
Recruit, designate, and train for leadership	**High**	**Moderate**	**Moderate**
Conduct educational meetings	High	High	**Low**
Develop educational materials	Low	High	Low
Create a learning collaborative	Moderate	**Moderate**	**Low**
Conduct educational outreach visits	Moderate	**High**	**Moderate**
Use Train the Trainer strategies	**High**	Moderate	**Moderate**
Conduct ongoing training	**Moderate**	Moderate	**Low**
Visit other sites	**Moderate**	**Moderate**	**Low**
Shadow other experts	Moderate	Moderate	**Moderate**
**Category 3: Data and Feedback**			
Facilitate relay of clinical data to providers	High	**Low**	**Moderate**
Use data experts	**High**	**Moderate**	**Moderate**
Capture and share local knowledge	**Moderate**	**Moderate**	**Moderate**
Develop and organize quality monitoring systems	High	**Low**	Moderate
**Category 4: Tailoring to Local Context**			
Assess for readiness and identify barriers and facilitators	**Moderate**	**Moderate**	Moderate
Tailor strategies	**High**	Moderate	**High**
Conduct local needs assessments	**High**	Moderate	Moderate
Promote adaptability	**High**	**Moderate**	Moderate
Develop a formal implementation blueprint	**High**	Moderate	Moderate
**Category 5: Technical and Financial Support**			
Centralize technical assistance	**High**	Low	Moderate
Access new funding	High	**Low**	**Moderate**
Fund and contract for clinical innovation	High	**Moderate**	Moderate
Develop resource sharing agreements	Moderate	**Low**	**Moderate**
**Category 6: System Changes**			
Change liability laws	Low	**Low**	Low
Revise professional roles	**High**	**Low**	Moderate
Create new clinical teams	High	Low	High

* Bold text indicates consensus was achieved in Round 1; non-bolded text indicates consensus was reached in Round 2
