# Supplementary figures and images for: Implementation Strategies to Enhance Safety-Net Hospitals’ Adoption of Screening, Brief Intervention, and Referral to Treatment for Opioid Use Disorder
Source: J Gen Intern Med. 2025 Aug 4;41(1):26–34. doi: 10.1007/s11606-025-09785-z (PMC12445236; doi:10.1007/s11606-025-09785-z)

**Appendix Figure 1.** Flowchart of the Delphi ranking process and timeline


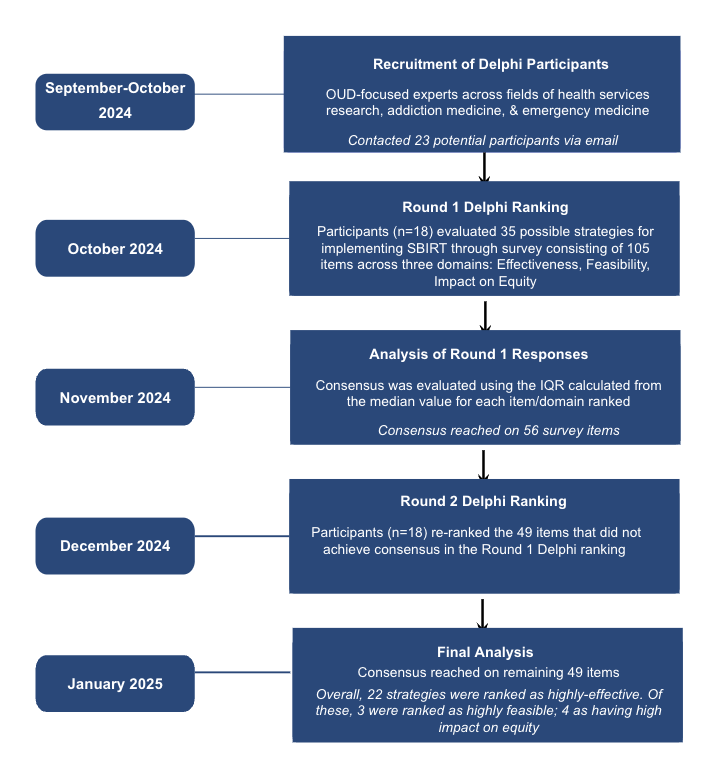

Supplement: Supplementary file 1 — Appendix Figure 1. Flowchart of the Delphi ranking process and timeline (DOCX 80.6 KB) [file 11606_2025_9785_MOESM1_ESM.docx]
